# Outcomes of Two-Staged Repair Procedures for Adult Penoscrotal Hypospadias: A Retrospective Analysis

**DOI:** 10.7759/cureus.99584

**Published:** 2025-12-18

**Authors:** Kawaljit Kaura, Taruna Singh, Mohd Altaf Mir, Abhay S Gaur

**Affiliations:** 1 Urology, All India Institute of Medical Sciences, Bathinda, IND; 2 General Surgery, University College of Medical Sciences, New Delhi, IND; 3 Burns and Plastic Surgery, All India Institute of Medical Sciences, Bathinda, IND; 4 Urology and Renal Transplant, All India Institute of Medical Sciences, Bathinda, IND

**Keywords:** adult hypospadias, hypospadias complications, hypospadias repair, penoscrotal hypospadias, proximal penile hypospadias

## Abstract

Objective: This study aimed to describe a two-stage repair technique (buccal mucosal grafting in Stage 1 and tunica vaginalis flap in Stage 2) and to evaluate its outcomes.

Methods: Ten cases of penoscrotal hypospadias with ventral chordee in adults were treated with two-stage repair (Group 1). Chordae correction was done in the first stage, a thin and unhealthy urethral plate was excised, and a buccal mucosal graft of 3-4 cm width was placed. The second stage was undertaken after three months, in which the neourethra was tabularized over a 14-Fr silicone catheter with the tunica vaginalis flap as the subsequent layer. Foley removal was done on postoperative day 5. The outcomes were compared with single-stage repair (Group 2) (n = 10) cases.

Results: While six cases had an excellent outcome in Group 1, only three had success in Group 2. Only one patient in Group 1 developed a urethrocutaneous fistula (UCF); Group 2 had five patients with UCF. In Group 1, one patient developed an infection with complete suture line disruption, and Group 2 had three cases each of wound infection and wound dehiscence. One patient in Group 1 and two in Group 2 developed superficial glans necrosis. The two-stage repair has a higher success rate than the single-stage repair (60% vs. 30%).

Conclusion: Staged repair and use of 14-Fr Foley and early Foley catheter removal improve adult penoscrotal hypospadias repair outcomes.

## Introduction

Most available information on the outcomes of hypospadias repair comes from the pediatric age group. Literature on adult hypospadias repair is limited.

The optimum timing for hypospadias surgery is at 6-18 months. This, however, is often not feasible in developing countries like ours. We often see untreated patients in adolescence or adulthood. Studies have associated repair of hypospadias performed later in life, such as in adolescence or adulthood, with poorer outcomes and increased complications [1]. This can be due to lesser availability of local tissue, poor vasculature, scarring from previous surgeries, or more frequent erections in adults in the immediate postoperative period (POD), leading to tension in suture lines [2-4]. Furthermore, the problem is of greater magnitude due to severe chordee in almost all adult cases. The meatus always shifts proximally after chordee correction, resulting in scrotal or perineal hypospadias in adults.

These problems have compelled us to modify our technique for hypospadias repair in adults. We hereby share our two-staged repair technique and its outcomes compared with single-stage repair in adult patients with adult penoscrotal hypospadias.

## Materials and methods

**Figure 1:**
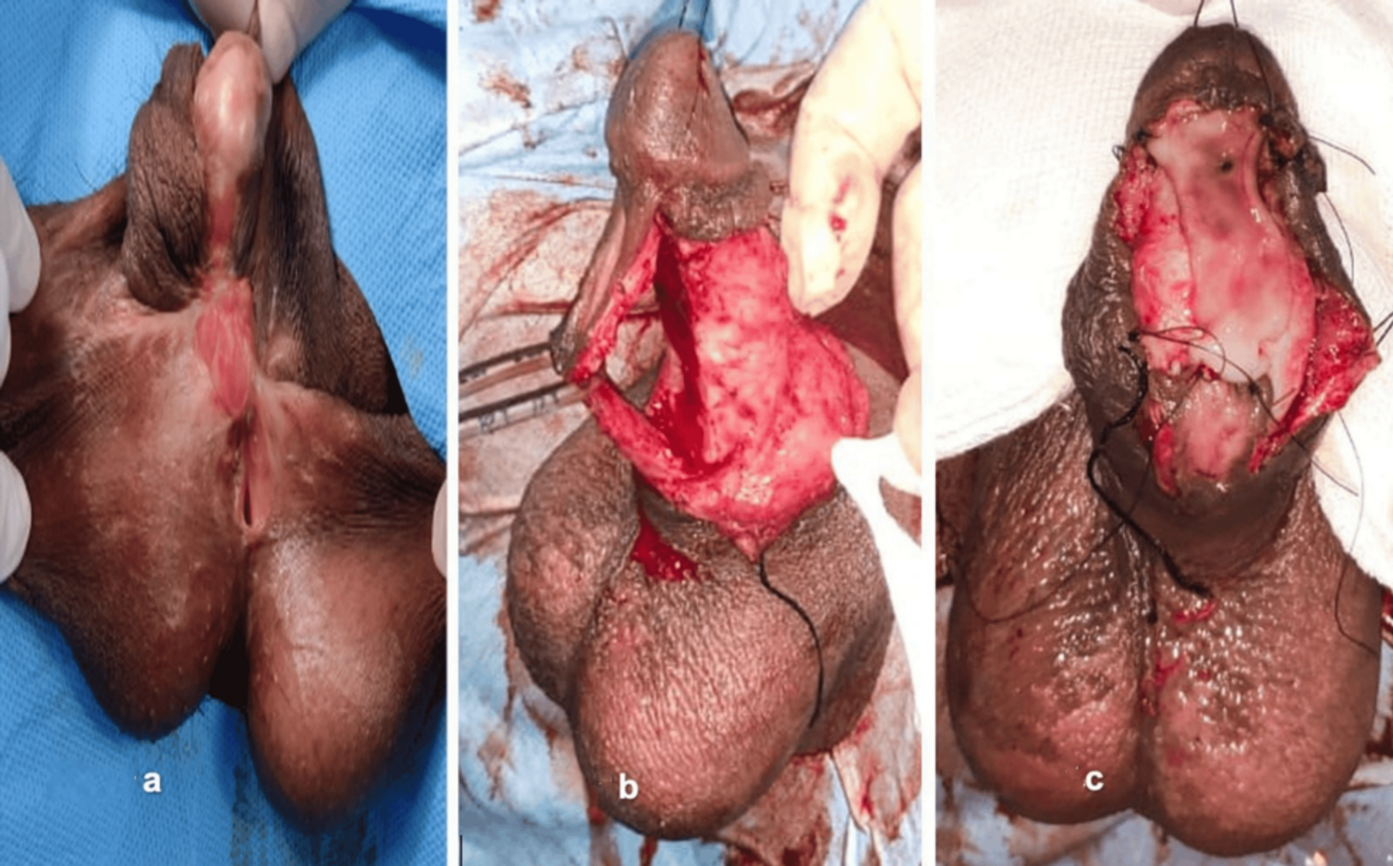
Hypospadias repair in hypospadias cripple with a penoscrotal hypospadias patient (a) Hypospadias cripple with penoscrotal hypospadias. (b) Degloving and mobilization of the urethral plate. (c) Placing a buccal mucosal graft

**Figure 2:**
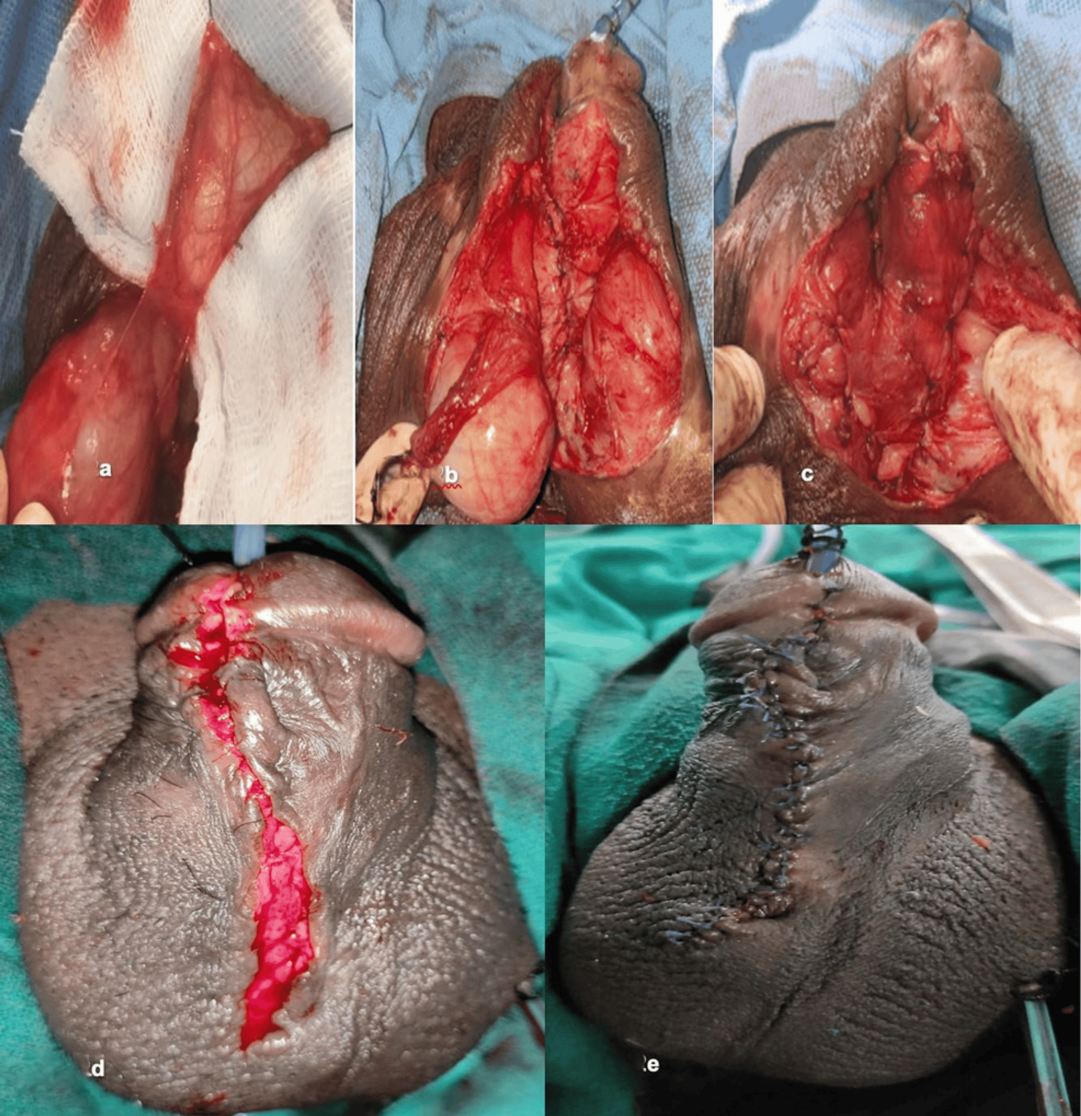
Hypospadias repair with tunica vaginalis flap (a) Tunica vaginalis graft from the testis. (b) Tunica vaginalis flap unstretched over the scrotum after harvest. (c) Appearance after completion of tunica vaginal graft placement over the neourethra and placing the testis back into the scrotum. (d) After subcutaneous tissue/dartos (third layer) closure. (e) After skin (fourth layer) closure

**Figure 3:**
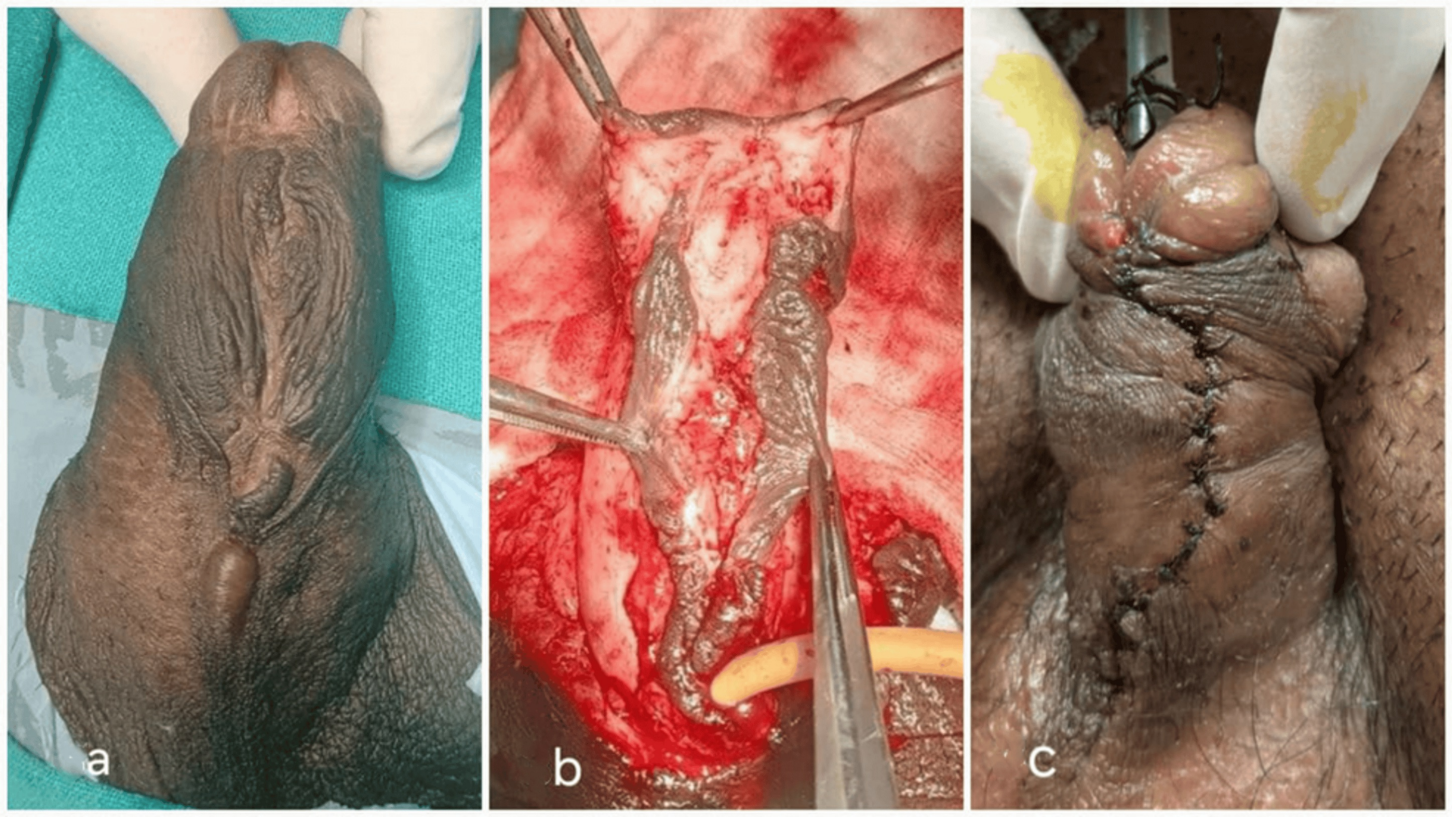
Single-stage repair was done similarly without the use of buccal mucosa graft (a) Single-stage repair (Group 2) penoscrotal hypospadias. (b) Incising the urethral plate with glans wings. (c) Tubularization of the urethral plate without placing a buccal mucosal graft and a tunica vaginalis flap

Ten consecutive cases of adult hypospadias with severe ventral chordee were evaluated. The location of the meatus, status of the urethral plate, degree of chordee, scarring of the surrounding tissues, details of previous surgery, and history of erectile function were noted. Two of the patients were hypospadias cripple, previously operated on at other centers. We compared the outcomes of two-staged repair (n = 10). Figures 1, 2 represent Group 1 with our single-stage repair (n = 10), and Figure 3 represents Group 2.

Details of our two-staged operative technique are as follows. The surgeries were performed under general anesthesia and in the lithotomy position. Preoperative antibiotics were given as routine.

Stage 1 repair

Chordee Correction

A thorough inspection was done. Gitte’s test was done to know the extent of ventral chordee. The extent of the scarring, if present, was noted. A circumcoronal incision was made, and penile degloving was done at the level of Buck’s fascia carefully, avoiding injury to neurovascular elements and the underlying corpora. The urethral plate was then dissected off the corpora starting proximally from the divergence of the corpora spongiosum (Figures 1a, 1b). Gitte’s test was repeated. The hypoplastic urethral plate was then transacted and excised along with surrounding scar tissue, if any. The deficient length was then measured.

Harvesting of Buccal Mucosa

Another team harvested the buccal mucosa to reduce operative time and avoid cross-contamination. A rectangular buccal mucosa graft of 3-4 cm width and required length was harvested from either cheek. The special precaution is that the incision is made away from the opening of the Stenson duct so as not to injure Stenson’s duct. The donor site was allowed to heal with secondary intention through mucolization from the margins.

Placing the Graft

The graft was then placed onto the spongiosum and sutured proximally to the distal end of the urethra and to the spongiosum with 5/0 Vicryl (Figure 1c). Quilting was done as deemed necessary. The urinary catheter was placed. The light dressing was done with wax gauze.

The patient was discharged on POD 3. The patient was advised to keep the area clean and moist until the next stage of repair.

Stage 2 repair

The second-stage repair was carried out three months after the first-stage repair.

Tubularization of Neourethral Plate

The contractures, developed due to scarring and shrinking of the graft, were released to avoid tension and get a 2-3 cm breadth of the urethral plate. The neourethral plate was then tubularized over a 14-Fr silicone Foley catheter using 5/0 polydioxanone interrupted sutures, creating a neourethra. Ventral dartos, if available, was used as the second layer for closure.

Harvesting the Tunica Vaginalis Flap

Near the old neourethra site wound, either testis was tunneled and delivered. A rectangular tunica vaginalis flap of sufficient length was then raised (Figure 2a). The flap was then placed over the neourethra as the third layer (Figures 2b, 2c). The flap was then sutured using 5/0 polydioxanone interrupted sutures. The testis was delivered back into the scrotum (Figure 2c).

Final Closure

Subcutaneous and skin were closed as the fourth layer in the usual manner (Figures 2d, 2e). The light dressing was done with wax gauze. The dressing was removed on POD 3, and the catheter was removed on POD 5. Antibiotics were administered postoperatively routinely for two weeks. All patients received oral diazepam postoperatively to prevent nocturnal erections. Single-stage repair was done similarly without the use of buccal mucosa graft, tunica vaginalis flap, and early removal of the Foley catheter (Figures 3a-3c).

Definition of a successful outcome

The successful outcome was defined as the distal location of the meatus and adequate urine flow with a flow rate >15 mL/second after six months of the surgery.

## Results

The mean age at presentation in Group 1 was 22.5 years, while in Group 2, it was 24.6 years. Group 1 had two cases in which patients had a proximally placed meatus despite the last surgery. One of these patients had undergone more than one surgery (Figure 1a). The other eight patients were primary cases.

**Figure 4:**
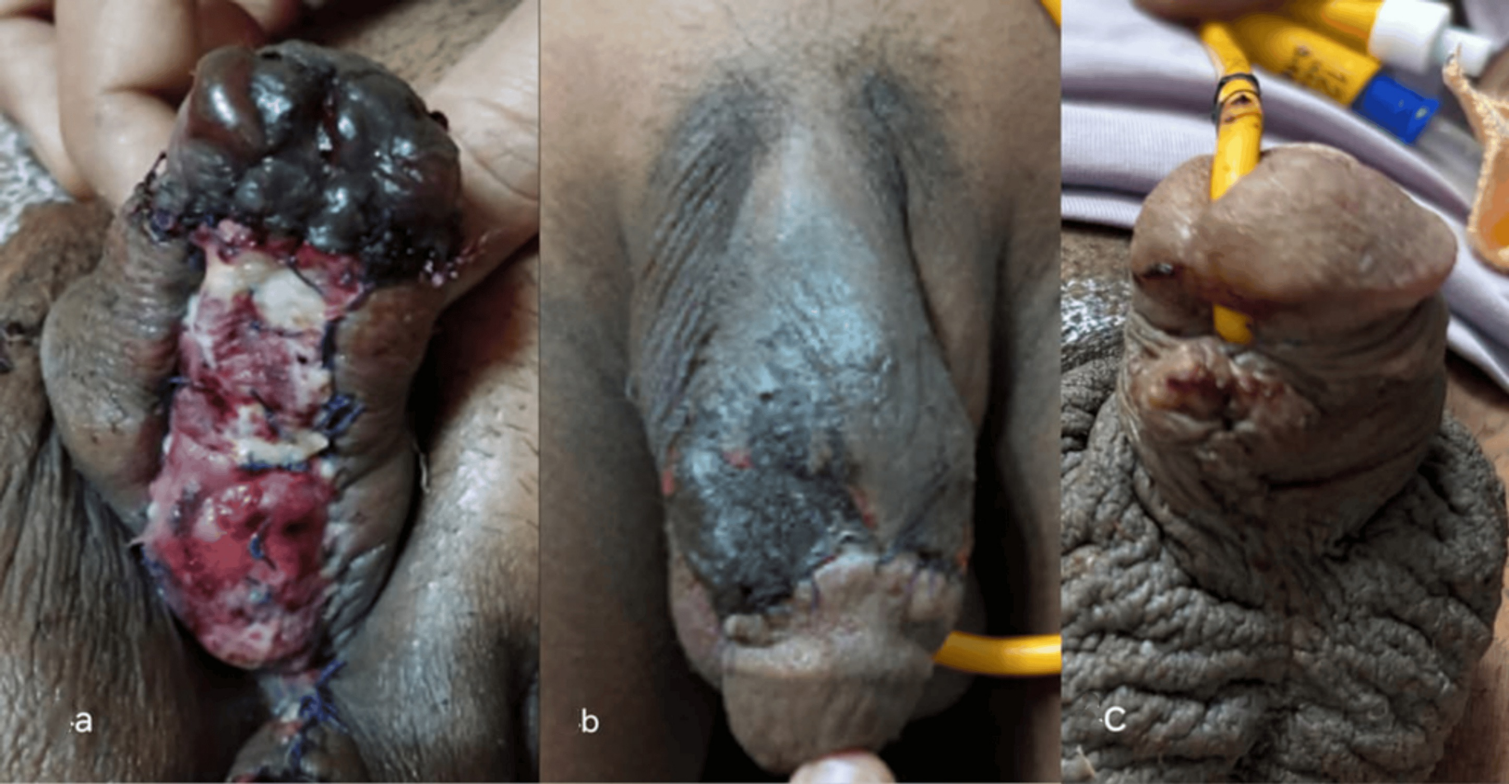
Complications after stage 1 repair (a) Superficial glans necrosis. (b) Superficial skin necrosis. (c) Urethrocutaneous fistula

Two patients developed complications after Stage 1 repair. Both of these patients were hypospadias cripples. Superficial glans necrosis was seen at POD 5 in one patient (Figure 4a).

The other patient developed superficial skin necrosis at POD 4 (Figure 4b). Both patients were managed with debridement and dressings, resulting in normal-looking tissue.

**Figure 5:**
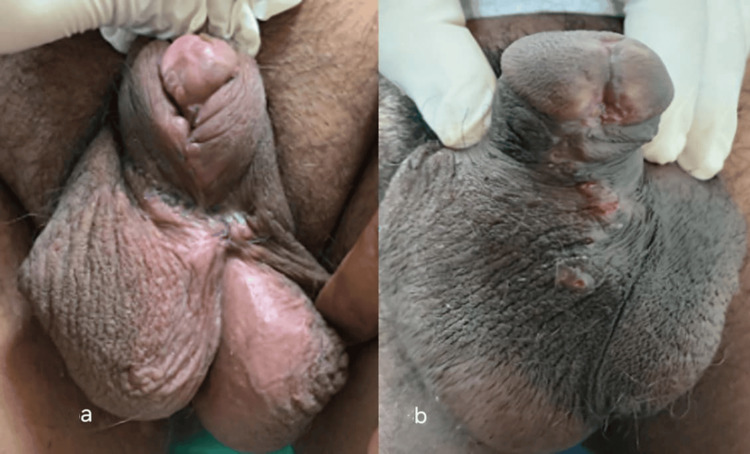
Outcome after stage 2 procedure (a) Appearance after six months of surgery in a hypospadias cripple. (b) Appearance after six months of surgery in another patient

After Stage 2 repair, complications were seen in two of 10 patients. One patient developed a wound infection with complete suture line disruption, while another developed a fistula. Six of 10 patients (60%) had good outcomes (Figures 5a, 5b).

One of them was a hypospadias cripple with previous multiple surgeries (Figure 5a), and the others were primary cases (Figure 4b). All six patients had straight penises with normal erections and good urinary flow with a directed stream while standing. Using a 14-Fr catheter did not lead to a narrowed urethral lumen as evidenced by uroflowmetry at three months and cystoscopic examinations six months postoperatively.

Group 2 had a success rate of 30%. Three patients had wound infections. Three patients had wound dehiscence. Urethrocutaneous (Figure 4c) fistula developed in five patients, and two had superficial glans necrosis. Demographics of cases are summarized in Table 1.

**Table 1:** Demographics of cases

Group 1			Group 2		
Case	Age (years)	Type	Case	Age (years)	Type
1	20	Primary	1	25	Primary
2	21	Re-do	2	24	Primary
3	22	Primary	3	27	Primary
4	23	Primary	4	22	Primary
5	24	Re-do	5	26	Primary
6	22	Primary	6	25	Primary
7	23	Primary	7	23	Primary
8	21	Primary	8	26	Primary
9	25	Primary	9	24	Primary
10	24	Primary	10	24	Primary

Description of a case in Group 1

Case 1: A 20-year-old patient had a proximally placed meatus despite the last surgery. This patient had undergone more than one surgery (Figure 1a).

Cases 2-4: These were primary cases.

Case 5: Patient had undergone primary surgery elsewhere.

Cases 6-10: These patients underwent primary repair.

Cases 1 and 5 developed complications after Stage 1 repair. Both of these patients were hypospadias cripples. Superficial glans necrosis was seen at POD 5 in Case 1 (Figure 4a). The Case 5 patient developed superficial skin necrosis at POD 4 (Figure 4b). Both patients were managed with debridement and dressings, resulting in normal-looking tissue.

After Stage 2 repair, complications were seen in two of 10 patients. One patient developed a wound infection with complete suture line disruption, while another developed a fistula. Six of 10 patients (60%) had good outcomes (Figures 5a, 5b). The final image of Case 1 is shown in Figure 5a. All six patients had straight penises with normal erections and good urinary flow with a directed stream while standing. Using a 14-Fr catheter did not lead to a narrowed urethral lumen as evidenced by uroflowmetry at three months and cystoscopic examinations six months postoperatively.

Description of a case in Group 2

Case 1: A 25-year-old male patient with uncorrected penoscrotal hypospadias (Figure 3a). He developed a urethrocutaneous fistula (UCF) at POD 4 (Figure 4c).

Cases 2-10: Three patients had wound infections. Three patients had wound dehiscence. UCF developed in five patients, and two had superficial glans necrosis.

## Discussion

The ultimate goals of hypospadias surgery are to give the patient a cosmetically acceptable penis, enabling him to micturate while standing and maintaining his fertility. These goals are readily achieved in pediatric patients, but in adults, they are often difficult to achieve [5]. Adult hypospadias repair has been associated with higher complications even in patients without prior repair [5]. Only one prospective study comparing adults and pediatric patients has reported 2.5 times higher complication rates in adults [6].

After previous repairs, patients with residual functional complications' hypospadias cripples pose greater challenges [7]. Superficial glans necrosis following hypospadias surgery has never been reported to the best of our knowledge. In hypospadias, there is an arrest of normal development of the corpus spongiosum and ventral prepuce [8]. The glans is the distal-most expansion of the corpus spongiosum [9]. Thereby, we assume that, in distal hypospadias, the blood supply of the glans is less developed and is more dependent on deep dorsal arteries. Previous surgeries render the tissue scarred, immobile, and hypovascular. Natural planes in such cases are lost, and dissection leads to damage to vascular supply, which could have been the cause of superficial skin and glans necrosis in our patients. It is also known that tight dressings can cause such complications, so we applied loose dressings on our patients.

The buccal graft contracture rate has been reported to be approximately 20% [10]. We predicted the same and used 3-4 cm in width grafts. Graft contracture seen in two patients was limited and did not require revision. None of the patients suffered from graft loss.

As three to six months are needed for neovascularization and adequate tissue healing, the Stage 2 repair was undertaken after three to six months. Recognizing that tissue pliability and healing are impaired in adults, infection rates are high, and learning from our previous experiences, we routinely obtain urinary culture preoperatively. Appropriate antibiotics were started prophylactically in the preoperative period and were continued till POD [10]. There are higher chances of hematoma formation and wound infection in penile urethroplasty. This, along with a deficiency of ventral dartos and vascularity, increases the risk of fistula formation [11]. Urinary extravasation, wound infection, and failure to provide vascularized coverage (waterproofing) of the tubularized neourethra are risk factors for UCF.

We used dorsal dartos, when sufficiently present, as a second layer to avoid overlapping sutures and the tunica vaginalis flap as a third layer to provide good waterproofing. Skin and subcutaneous tissue were then closed.

We also changed our practice and used small-caliber (14 Fr) silicone catheters. This was done so as not to occlude the lumen of the urethra, allowing the infected materials and secretions to drain freely along the catheter. The patients were asked to gently massage their penis from the base to the tip of the penis to express the collection.

We also advocate the early removal of urinary catheters on POD 5, contrary to usual practices. This was based on our observation from Group 2 patients, where fistula and wound dehiscence started from Days 12-15 of POD, and all of these patients did well until PODs 7-10. Early Foley removal reduces catheter-induced trauma on edematous tissues and the tension produced due to nocturnal erections. Also, wound contraction sets in by this time, leading to tension on suture lines. The risk of catheter-associated urinary tract infections is also reduced, further decreasing the risk of complications.

Although the role is limited, all the patients received oral diazepam at bedtime to reduce nocturnal erections. Using a 14-Fr catheter did not lead to a narrowed urethral lumen as evidenced by uroflowmetry at three months and cystoscopic examination at six months postoperatively.

The success rate with our two-staged technique was 60%. Secrest et al. managed 190 patients, 1-58 years old (average 16 years) with complications of hypospadias repair, and were the first to report a success rate of 94.4%. However, the success rate was 45% among those requiring urethral reconstruction [12]. Later, Li et al., in a large series of 113 adolescent and adult patients, reported success and low complications using bladder mucosa graft [13]. We have no experience with bladder mucosa grafts and have used only buccal mucosa grafts. Although Hensle et al. reported an 88.1% success rate, the complication rate was 63.6% [5]. They used multiple techniques and graft types for repair, and only nine of 42 patients had a proximal meatus. In a study of 88 patients, Hensle et al. reported a 89.6% success rate, with only two of 88 patients having proximal hypospadias [5]. Adayener and Akyol reported a 91.3% success rate in 119 patients, all of whom had distal hypospadias [14]. Our lower success rate could be due to all patients having proximal meatus and two (20%) being hypospadias cripple. It is known that the success rate of hypospadias decreases with the proximal location of the meatus and the number of previous surgeries. A recent cross-sectional study from Switzerland reported that the shape and position of the meatus are the least important penile aspects for women [15]. A reconstructed penis after hypospadias surgery with distal hypospadias was considered similar to the circumcised penis in appearance [16]. The patients with good outcomes denied the need for further surgery for cosmesis, as they had acceptable results. Our study has limitations like a small sample size and a short follow-up. However, a large sample size for such a rare disease and longer follow-up for such complex and multistage reconstruction is always difficult.

However, limitations are the retrospective nature and small sample size of our study. It needs further strengthening of evidence base through prospective studies and randomized controlled trials.

## Conclusions

Proximal hypospadias repair in adults is challenging, especially in cases with previous attempts at unsuccessful repair, and the results are dismal. We report a 60% success rate. We recommend staged repair using buccal mucosal graft, vascularized flaps such as tunica vaginalis flap, layered closure, smaller caliber catheters, and early catheter removal to decrease the risk of complications and improve the outcomes.

## References

[1] van der Horst HJ, de Wall LL (2017). Hypospadias, all there is to know. Eur J Pediatr.

[2] Barbagli G, De Angelis M, Palminteri E, Lazzeri M (2006). Failed hypospadias repair presenting in adults. Eur Urol.

[3] Aldamanhori RB, Osman NI, Inman RD, Chapple CR (2018). Contemporary outcomes of hypospadias retrieval surgery in adults. BJU Int.

[4] Vricella GJ, Coplen DE (2016). Adult hypospadias: urethral and penile reconstruction. Curr Opin Urol.

[5] Hensle TW, Tennenbaum SY, Reiley EA, Pollard J (2001). Hypospadias repair in adults: adventures and misadventures. J Urol.

[6] Bhat A, Bhat M, Kumar V, Kumar R, Mittal R, Saksena G (2016). Comparison of variables affecting the surgical outcomes of tubularized incised plate urethroplasty in adult and pediatric hypospadias. J Pediatr Urol.

[7] van der Werff JF, van der Meulen JC (2000). Treatment modalities for hypospadias cripples. Plast Reconstr Surg.

[8] Baskin LS, Ebbers MB (2006). Hypospadias: anatomy, etiology, and technique. J Pediatr Surg.

[9] Yiee JH, Baskin LS (2010). Penile embryology and anatomy. ScientificWorldJournal.

[10] Myers JB, McAninch JW, Erickson BA, Breyer BN (2012). Treatment of adults with complications from previous hypospadias surgery. J Urol.

[11] Mundy AR (2006). Failed hypospadias repair presenting in adults. Eur Urol.

[12] Secrest CL, Jordan GH, Winslow BH, Horton CE, McCraw JB, Gilbert DA, Devine CJ Jr (1993). Repair of the complications of hypospadias surgery. J Urol.

[13] Li LC, Zhang X, Zhou SW, Zhou XC, Yang WM, Zhang YS (1995). Experience with repair of hypospadias using bladder mucosa in adolescents and adults. J Urol.

[14] Adayener C, Akyol I (2006). Distal hypospadias repair in adults: the results of 97 cases. Urol Int.

[15] Snodgrass W, Villanueva C, Bush N (2014). Primary and reoperative hypospadias repair in adults--are results different than in children?. J Urol.

[16] Ruppen-Greeff NK, Weber DM, Gobet R, Landolt MA (2015). What is a good looking penis? How women rate the penile appearance of men with surgically corrected hypospadias. J Sex Med.

